# Metastatic Potential of Very Small (≤2 cm) Renal Cell Carcinoma: Insights from a Single-Center Experience and Review of the Literature

**DOI:** 10.3390/jcm14196781

**Published:** 2025-09-25

**Authors:** Lorenzo Giuseppe Luciani, Tommaso Ceccato, Tommaso Cai, Stefano Chiodini, Simone Botti, Valentino Vattovani, Marco Puglisi, Andrea Abramo, Daniele Mattevi

**Affiliations:** 1 Robotic Surgery Unit, Santa Chiara Regional and Teaching Hospital, 38122 Trento, Italy; 2 Urology Division, Santa Chiara Regional and Teaching Hospital, 38122 Trento, Italy; 3 Centre for Medical Sciences, University of Trento, 38122 Trento, Italy

**Keywords:** renal cell carcinoma, small, synchronous, metastases, treatment, robotic-assisted partial nephrectomy, RAPN

## Abstract

**Background/Objectives:** Small renal masses (SRMs) are being detected more often due to the increasing use of imaging techniques. Many of these lesions are benign or grow slowly, but a small proportion can exhibit aggressive behavior. Several reports have shown that synchronous metastases may occur even in small renal cell carcinomas (RCCs). Our aim is to assess the malignant potential and the metastatic risk of very small RCCs (≤2 cm). **Methods:** We reviewed consecutive patients who underwent partial nephrectomy for SRMs at our tertiary referral center between 2005 and 2024, focusing on those with a maximum pathologic diameter ≤ 2 cm. Clinical and pathological data were collected, and cases with aggressive features were described. In addition, a literature search on the Medline/PubMed database was performed to identify previously published cases of RCC ≤ 2 cm and to assess their risk of synchronous metastases (SM). **Results:** Among 578 patients who underwent partial nephrectomy, 116 patients (20%) had tumors ≤ 2 cm, 90 (77.5%) of which were malignant, whereas 22.5% were benign (oncocytoma = 13%; angiomyolipoma = 5%). Median age and tumor size were 51 yrs and 1.7 cm, respectively. Histology showed clear cell (72.2%), papillary (20%), chromophobe (6.6%), and mixed (0.9%). Two patients (2.2%) experienced aggressive disease: one with synchronous metastases and one with recurrence and later progression. From the literature, we identified 16 additional cases of RCC ≤ 2 cm with synchronous metastases and found an important heterogeneity of results regarding the metastatic potential of SRMs. **Conclusions:** Although uncommon, synchronous metastases can occur in RCCs even smaller than 1–2 cm. Reported rates for SM of SRMs across the literature range between 1% and 13%, with higher risk observed in tumors larger than 3 cm, but without an absolute safe cutoff. Tumor size alone is therefore insufficient to exclude aggressive potential. Clinical decision-making should consider histology, grade, patient age, radiologic features, and emerging molecular markers to guide surveillance and treatment in this growing patient population.

## 1. Introduction

In recent years, there has been renewed interest in small renal masses (SRMs). These entities are defined as clinical stage T1a solid renal lesions measuring ≤4 cm with contrast enhancement at the CT scan (described as an increase of at least 15 Hounsfield units (HUs) in the tumor site) [1]. This interest has evolved alongside the development of minimally invasive approaches to treat (ablative techniques) or not to treat (active surveillance and watchful waiting) these incidental SRMs in frail patients, with impaired renal function, or in individuals with significant comorbidities who are either unfit or decline conventional surgery. Despite these new approaches, for cT1a lesions, robotic nephron-sparing surgery (RAPN) is regarded as the standard therapeutic option in patients who are suitable surgical candidates and in whom the procedure is technically feasible [2].

Established features of SRMs include a benign diagnosis in about 25% of cases, a probability of malignancy increasing with size [3,4,5,6,7], a low growth rate defined in terms of 1–3 mm per year [6,8], and a low risk of metastatic spread (metachronous metastasis) described in the round of 1–3% [6,8,9]. These characteristics suggest the possibility of a conservative/less invasive management of these incidental entities, whose number is constantly rising due to the spreading of imaging techniques.

Nevertheless, at present, conclusive and robust evidence that allows for the exclusion of metastatic potential/aggressive behavior of a lesion on the basis of tumor size alone is lacking, with several studies underlying aggressive features of these SRMs with the possibility of synchronous metastasis [10,11,12,13,14,15,16].

Our objective is to assess the aggressive potential of very small renal cell carcinoma (RCC) and its current management.

## 2. Materials and Methods

We retrospectively reviewed consecutive patients who underwent partial nephrectomy in our tertiary referral center for small RCC from January 2005 to 2024 using a prospectively maintained database. The analysis was restricted to patients with a tumor whose maximum pathologic diameter was less than or equal to 2 cm (≤2 cm). Cases with aggressive potential are described in detail. Demographics, perioperative, and follow-up oncological outcomes were collected and analyzed. We subsequently performed a review of the literature on the Medline/PubMed database using a free-text search strategy (“small” AND “metastatic” AND “renal cell carcinoma”; “renal mass” AND “metastasis” AND “tumor size”). Only cases with a tumor diameter ≤2 cm and clinical information fully available were considered. Studies were initially screened by title and abstract; when these were insufficient to establish eligibility, the full text was retrieved and assessed against the inclusion and exclusion criteria. A systematic or meta-analytical comparison falls outside the scope of this work.

## 3. Results

During the study period, 578 partial nephrectomies were performed, including 431 robot-assisted procedures. A total of 116 of 578 (20%) had a tumor diameter ≤ 2 cm, in which 90 (77.5%) of 116 were malignant. Median age at surgery was 51 years old and median tumor size was 1.7 cm. The Fuhrman nuclear grading was G1 in 33, G2 in 37, and G3 in 8, not applicable in 6. Histology showed clear cell, papillary, chromophobe, and mixed RCC in 65, 18, 6, and 1 cases, respectively. Over follow-up, 2 (2.2%) of 90 patients experienced an aggressive disease: one case with synchronous metastases (case A), and one with progression (case B).

### 3.1. Case A: Presentation and Management

**Figure 1:**
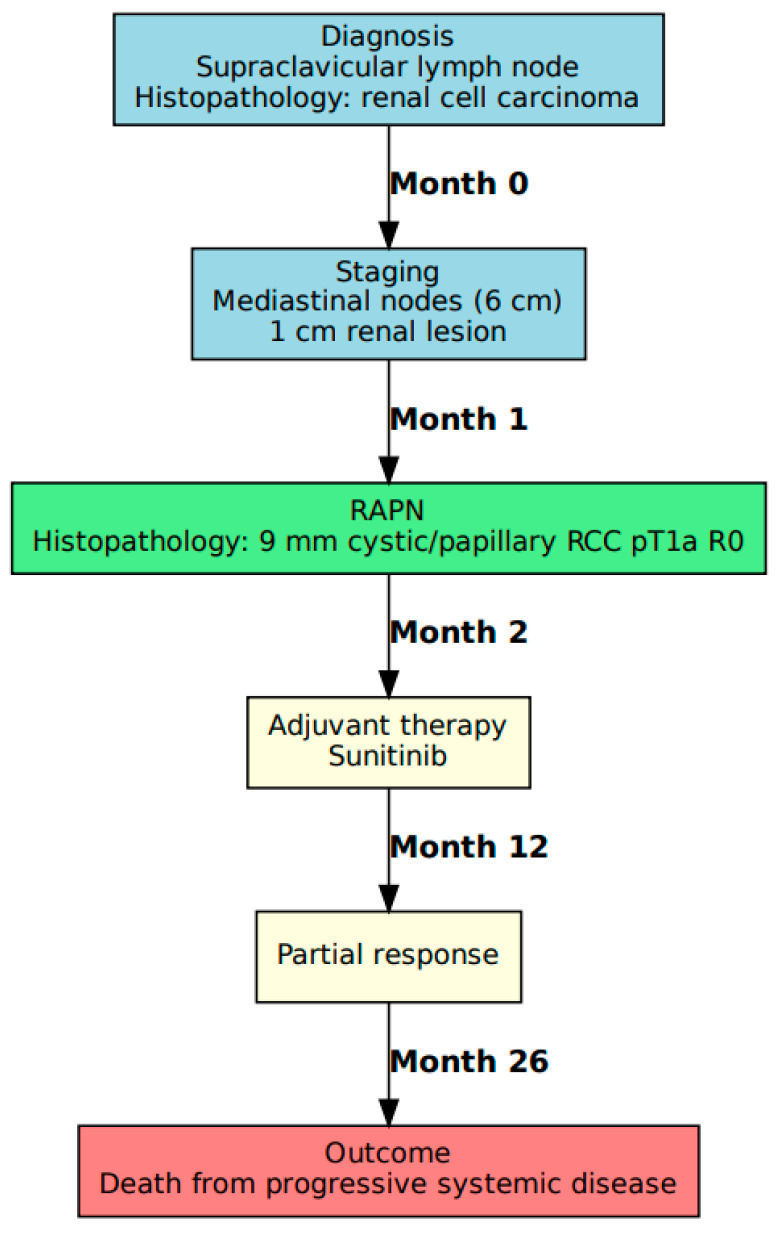
Case A flowchart.

A 35-year-old female presented with left supraclavicular lymph node enlargement. An excisional biopsy performed in an outside hospital revealed a metastatic RCC. Staging CT scan and MRI showed multiple enlarged mediastinic lymph nodes (the largest of which measured 6 cm), and a 1 cm area with altered density at the lower pole of the left kidney. Due to its medial position, the mass was unsuitable for a percutaneous biopsy. After a multidisciplinary consultation, the patient underwent a transperitoneal robotic-assisted partial nephrectomy (RAPN) of a 1 cm intrarenal solid mass with intraoperative ultrasound guidance to confirm the renal origin of the malignancy. The postoperative course was uneventful. Histopathology confirmed a 9 mm cystic and papillary RCC with negative margins. The patient underwent adjuvant therapy with sunitinib with a transient partial response at 12 months and died at 26 months postoperatively with progressive systemic disease (Figure 1).

### 3.2. Case B: Presentation and Management

**Figure 2:**
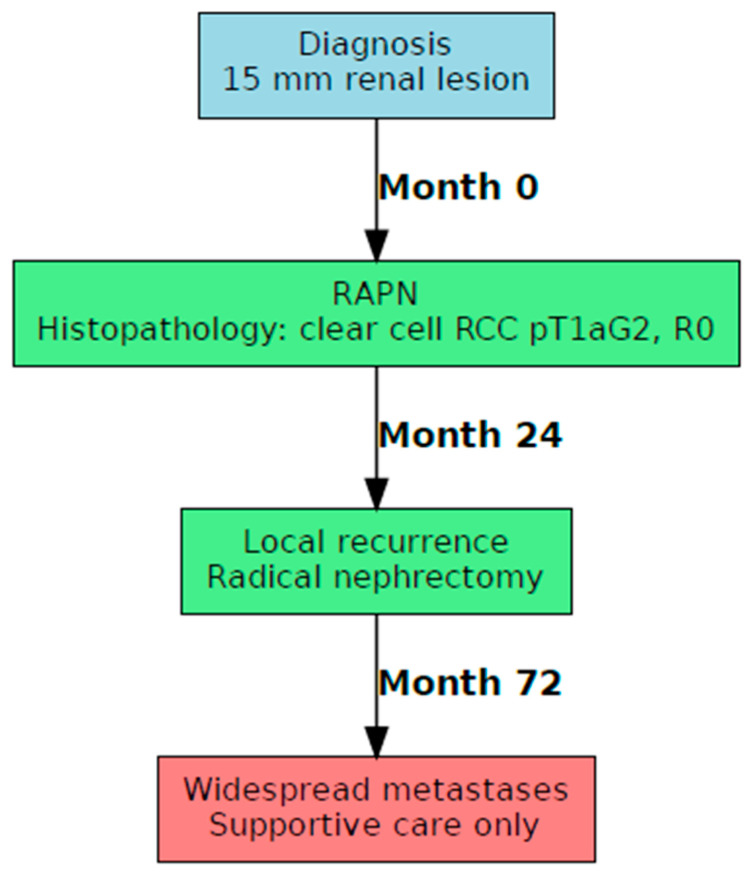
Case B flowchart.

A 75-year-old woman, with an incidental left renal mass of 15 mm and no significant comorbidities or previous surgical procedures in her clinical history, underwent a transperitoneal RAPN with intraoperative ultrasound guidance. Final histopathology demonstrated a pT1a, grade 2, clear cell renal carcinoma with negative margins. Two years later, local recurrence occurred and was treated with radical nephrectomy. Six years after the first procedure, widespread metastatic disease developed and was managed with best supportive care (Figure 2).

### 3.3. Review of the Literature

Most studies indicate that the increasing size of RCC tumors is associated with a higher probability of synchronous metastases (SM) and worse survival (Table 1). In particular, multiple surgical series, both single institution and multi-centered, show an increased rate of SM, ranging from 0% in RCC with a tumor size < 1.0 cm, to 1.1–13.8% in RCC of 3–4 cm.

The data available are heterogeneous and do not allow one to define a threshold under which the risk of concomitant metastatic disease is negligible. However, it can be concluded that a very small renal lesion can hide an aggressive behavior at the time of diagnosis.

In Table 2, we summarized and focused on the cases of renal cell carcinoma < or equal to 2 cm associated with synchronous metastases, where presentation (incidental/symptomatic), size of the primary tumor, hystotype, metastatic site, therapy, and oncologic outcomes are reported in detail.

## 4. Discussion

In recent decades, the incidence of new small renal masses has increased due to the widespread use of imaging techniques [38]. The small size of these entities and their lower metastatic potential have led to conservative management in specific cohorts of patients. In this review, we have collected evidence from institutional series, multicenter cohorts, population-based registries, and autopsy studies, focusing on the risk of synchronous metastases in SRMs. The search has revealed a considerable heterogeneity of data that cannot lead to strong evidence.

Across the studies analyzed, the rate of SM in SRMs ranged from <1% in highly selected surgical cohorts to nearly 13% in population-based analyses.

Remzi et al. reported on 287 patients with tumors ≤ 4 cm and found SM in 2.4% of tumors < 3 cm compared to 8.4% of those 3–4 cm, suggesting that the aggressive potential of small renal cell carcinoma increases dramatically beyond this threshold [7]. Similarly, Ingimarsson et al. described an SM rate of 11% for tumors till 4.0 cm and of 25% for tumors > 4 cm [19], Gudmundsson et al. reported a SM rate of 5% in tumor smaller than 3 cm and of 10% in those of 3–4 cm [14] and Kunkle et al. [10] highlighted an increase in terms of rate of SM between tumor of 3–4 cm (12%) and of 4–5 cm (21%).

Reporting a single-center series of 1290 patients who underwent a radical nephrectomy or nephron-sparing surgery, Pahernik et al. found similar evidence. In particular, their data showed that the risk of adverse pathological features and poorer oncological outcomes increases progressively with tumor diameter above 2 cm. In fact, under this size the risk of SM was 2.9%, increasing subsequently to 7.5% and 9.1% for tumors, respectively of 2.1–3.0 cm and 3.1–4.0 cm. However, the size was also correlated to more advanced pathological stage, higher grade, and less favorable long-term outcomes, supporting the TNM system [15].

Moreover, these findings were confirmed by several retrospective analyses of the SEER (Surveillance, Epidemiology, and End Results) database. Nguyen et al., analyzing 24,253 patients, described a sigmoidal relationship between tumor size and both metastatic presentation at diagnosis and cancer-specific mortality, with a steep rise beginning around 3–4 cm. In particular, they described a risk of SM of 7.4% and 11.8% for 3.1–4.0 cm and 4.1–5.0 cm lesions, respectively [12]. Similar data come from the SEER analysis conducted by Lunghezzani et al. [11] and Hellenthal et al. [17]. They studied the relationship between tumor size and synchronous metastasis in 22,204 and 56,011 patients, respectively. Their findings coincide with what was already established by previous studies, confirming the direct increase of SM rate with the increase of tumor size and underlining that even SRMs are at risk for SM, especially patients with clear cell renal cell cancer.

What differs slightly from the data presented are the findings from the autoptic series described in 1998 by Wunderlich et al., comparing tumor size to the extent of disease in 260 cases of RCC out of 14,793 autopsies. When 104 tumors smaller than 4 cm were analyzed, a significant increase in the incidence of detected distant metastases was seen as primary tumor diameter increased from 2 cm or smaller (12.1%) to 2.1 to 3 cm (21.4%) to 3.1 to 4 cm (48.8%). These data were likely influenced by methodological biases of the time and conflicted significantly with other studies indicating a much lower rate of metastasis in small RCC [39]. Nonetheless, a high heterogeneity of the available data is evident.

On the other hand, a series by Ku et al. demonstrated an association between the tumor size and the risk of metachronous metastasis, but a significant correlation with SM was not found [40]. Similarly, Klatte et al. reported that tumor size was not a predictor of synchronous metastatic disease and, describing a 7% prevalence of metastasis in 1208 patients undergoing nephrectomy for renal masses 1 cm or less, confirmed that it is not possible to identify a tumor size safety threshold.

Likewise, SEER studies recruiting over 100,000 cases altogether showed a significant proportion of RCC < 1 cm presenting with SM: from 1.4 to 4.8% [11,12,17,25,26].

As a matter of fact, in our series, a 2.2% rate of RCC—measuring 9 and 15 mm—had an adverse outcome, underlining again that an aggressive biology might be present even in a few millimeters of tumor and that the chance of facing a very small and aggressive RCC should be considered in urologic practice.

Including our case described above, 16 cases of RCC ≤ 2 cm with synchronous metastases have been reported in the literature, 6 of which were autoptic findings (Table 2). The case we report concerns the smallest renal carcinoma (9 mm) with metastases diagnosed by imaging and treated with robotic partial nephrectomy and targeted therapy. The cases reported by Aizawa et al., about a 74-year-old man with 8 mm RCC with symptomatic bone metastases [28] and by Talamo et al., about a 51-year-old man with a 9 mm RCC with widespread metastases to the lungs, lymph nodes, and vertebrae, were both cases in which the primary tumor was found only at autopsy [37]. The most common histotypes were clear cell and papillary carcinomas, and the metastatic sites were unpredictable. Of note, a relatively higher rate of papillary RCC was observed in our series of very small RCC (20% vs. 13–20%), as compared to renal masses irrespective of their size [2]. The management of these patients remains controversial. A variety of treatments have been performed, from radical or partial nephrectomy with or without adjuvant targeted therapy to best supportive care alone. Although survival in this case was longer than in the other reported cases, the prognosis remains poor despite the small size of the renal primary tumors and irrespective of the treatment strategy, except in an incidentally detected disease with a limited metastatic burden.

In these cases of metastatic disease originating from an SRM, Tappero et al. and Di Natale et al. have recently demonstrated the association between cytoreductive nephrectomy and higher OS [41,42,43].

Future perspectives include parameters to be used in a predictive model that can better distinguish the indolent behavior or the metastatic potential of the SRMs. For example, imaging parameters (e.g., Ktrans and Kep) and tumor radiological characteristics (e.g., pseudocapsule) may enable gene-based risk stratification in small RCC, being associated with RNA expression as poor prognostic factors [44]. Another promising field is miRNA biomarkers that may differentiate between non-progressive ccRCC tumors and those that progress to metastatic disease in the context of SRMs [45].

Our review has several limitations, basically linked to the kind of data reported in the literature. First of all, most results were extrapolated from retrospective population-based studies with diagnoses recorded with codes. As recognized by the authors, there could be a bias due to the lack of a central revision of staging and pathology, or misinterpretation or incorrect coding in the register. On the other hand, the autopsy series reflects a very different patient population and older diagnostic practices, while the surgical series may underestimate the true risk because only operable patients were included. Moreover, in surgical series, there may be a bias linked to a selection of patients depending on the type of hospital (referral center or not), and some data may be affected by the inclusion of benign tumors. This heterogeneity explains the conflicting results across the literature and emphasizes the need for prospective, standardized studies.

## 5. Conclusions

The risk of synchronous metastasis in SRMs is low, but real. Several studies suggest rates between 1% and 13%, with a tendency toward higher risk in tumors larger than 3 cm. However, no size threshold completely excludes the possibility of metastatic disease. Tumor diameter remains a useful clinical parameter, but size alone cannot reliably distinguish indolent from aggressive disease, and other features have to be considered in the management of these lesions: histological subtype, tumor grade, and patient age. Better integration of clinical, pathological, radiological, and molecular predictors is needed to refine risk stratification and to guide choices between surveillance and surgical treatment. A small RCC should be kept in mind in case of widespread metastases without an evident primitive tumor.

## Figures and Tables

**Table 1:** Incidence rate of RCC with synchronous metastases (SM) according to the size of the primary tumor.

Author	Type of Study	<1.0	1.1–2.0	2.1–3.0	3.1–4.0	4.1–5.0	5.1–6.0	6.1–7.0
Remzi [7]	Single-center, retrospective	2.4%			8.4%	NR	NR	NR
Thompson [9]	Single-center, retrospective	0%	0%	0.2%	1.8%	2.3%	6.8%	6.6%
Kunkle [10]	Single-center, retrospective	0%	0%	10%	12%	21%	28%	39%
Lughezzani [11]	SEER database	4.8%	4.2%	4.9%	7.1%	12.1%	13.3%	18.4%
Nguyen [12]	SEER database	1.4%	2.5%	4.7%	7.4%	11.8%	15.9%	21.6%
Klatte [13]	Multi-center, retrospective	7%	6%	5%	8%	NR	NR	NR
Gudmundsson [14]	Population based	0%	4%	5%	10%	14%	15%	28%
Pahernik [15]	Single-center, retrospective	3%		2.6%	6.0%	NR	NR	NR
Lee [16]	Single-center, retrospective	0%	0.5%	1.2%	1.4%	NR	NR	NR
Hellenthal [17]	Retrospective (SEER)	4%	4%	5%	7%	11%	13%	18%
Umbreit [18]	Single-center, retrospective	0%	0%	0.2%	1.1%	2.9%	4.1%	5.5%
Ingimarsson [19]	Population-based	0%	11%			25%		
Pahernik [20]	Single-center, retrospective	2.9%		7.5%	9.1%	NR	NR	NR
Steffens [21]	German Renal Tumor Network	2.3%		2.4%	3.0%	NR	NR	NR
Miller [22]	Single-center, retrospective	0%			13.8%	NR	NR	NR
Zastrow [23]	Multi-center retrospective	1.6%			2.8%	3.3%	3.8%	7.0%
Zu [23]	Single-center, retrospective	2.1%		1.6%	0%	NR	NR	NR
Pecoraro [24]	Retrospective (SEER)	NR	0.7%	1%	1.9%	3%	5.1%	7.4%
M Monda [25]	Retrospective (SEER)	2.7%		2.5%	4%	7.1%	11.5%	16.7%
Kates [26]	Retrospective (SEER)	2.4%		4.3%	NR	NR	NR	NR
**Range**		**0–7**	**0–6**	**0.2–10**	**1.1–13.8**	**2.3–21**	**4.1–28**	**5.5–39**

**Table 2:** Case report and case series of RCC equal to or <2 cm with synchronous metastases.

Author	Presentation	Primary Tumor Size (mm)	Hystotype	Metastatic Site	Therapy	Outcome (mos)
Ishii [27]	Incidental	20	clear cell	hilar lymph nodes	open partial nephrectomy + INF	ANED (18)
Aizawa [28]	Incidental *	20	papillary	bone	BSC	DNED (4)
Dong [29]	Symptomatic	18	sarcomatoid clear cell	bones, lymph nodes, adrenals, and liver	Laparoscopic parzial nefrectomy	NR
Hwang [30]	Symptomatic	18	clear cell	sigmoid, liver, lung, etc.	BSC	DOD (3)
Alam [31]	Symptomatic	16	clear cell	bone	BSC	NR
Chalfin [32]	Symptomatic	16	clear cell	brain, lung	BSC	NR
Aizawa [28]	Symptomatic *	15	clear cell	bone	BSC	DOD (3)
Curry [33]	Symptomatic	15	tubular adenocarcinoma	supradiaphragmatic lymph nodes	radical nephrectomy	DOD (30)
Kume [34]	Symptomatic	15	clear cell + sarcomatoid	bone	embolization	DOD (27)
Kume [34]	Symptomatic	13	clear cell + sarcomatoid	bone	embolization	DOD (6)
Evins [35]	Symptomatic *	13	papillary	lungs, bone marrow, lymph nodes	orchiectomy + DES	DOD (6)
Masago [36]	Incidental	13	clear cell	pancreas	radical nephrectomy	ANED (12)
Aizawa [28]	Symptomatic *	12	sarcomatoid	chestwall	BSC	DOD (3)
Talamo [37]	Symptomatic *	9	clear cell	lungs, bones, lymph nodes	BSC	DOD (2)
Present case	Symptomatic	9	papillary	supradiaphragmatic lymph nodes	RAPN + targeted therapy	DOD (26)
Aizawa [28]	Symptomatic *	8	clear cell	bone	BSC	DOD (7)

* indicates autoptic diagnosis. DOD: dead of disease; DNED: dead no evidence of disease; ANED: alive no evidence of disease; AWD: alive with disease; RAPN: robotic-assisted partial nephrectomy, BSC: best supportive care.

## Data Availability

The original contributions presented in this study are included in the article. Further inquiries can be directed to the corresponding author(s).

## Abbreviations

The following abbreviations are used in this manuscript:

RCC	Renal Cell Carcinoma
RAPN	Robotic-assisted partial nephrectomy
BSC	Best Supportive care
SM	Synchronous Metastases
SRMs	Small Renal Masses
